# Shifts in the ESR Spectra of Alkali-Metal Atoms (Li, Na, K, Rb) on Helium Nanodroplets

**DOI:** 10.1002/cphc.201200697

**Published:** 2012-11-04

**Authors:** Andreas W Hauser, Thomas Gruber, Michael Filatov, Wolfgang E Ernst

**Affiliations:** [a] Institute of Experimental Physics, Graz University of TechnologyPetersgasse 16, 8010 Graz (Austria); [b] Mulliken Center for Theoretical Chemistry, Institut für Physikalische und Theoretische Chemie, Universität BonnBeringstrasse 4, 53115 Bonn (Germany)

**Keywords:** alkali-metals, density functional calculations, electron spin resonance, nanodroplets, relativistic coupled-cluster theory

## Abstract

He-droplet-induced changes of the hyperfine structure constants of alkali-metal atoms are investigated by a combination of relativistically corrected ab initio methods with a simulation of the helium density distribution based on He density functional theory. Starting from an accurate description of the variation of the hyperfine structure constant in the M–He diatomic systems (M=Li, Na, K, Rb) as a function of the interatomic distance we simulate the shifts induced by droplets of up to 10 000 ^4^He atoms. All theoretical predictions for the relative shifts in the isotropic hyperfine coupling constants of the alkali-metal atoms attached to helium droplets of different size are then tied to a single, experimentally derived parameter of Rb.

## 1. Introduction

Recently, a new spectroscopic technique has been developed that allows for a detection of electron spin resonance (ESR) transitions in doped helium nanodroplets.^1^, ^2^ It has the potential to monitor interactions between dopants sitting on the surface and inside a helium droplet of a given size.^3^ In ref. 4 our group presented hyperfine-resolved spectra of ESR transitions of rubidium atoms attached to superfluid helium nanodroplets. The high sensitivity of this experimental technique led to the observation of hyperfine shifts in the ESR spectra caused by the chemically inert but physically disturbing helium environment: shifts of the well-known hyperfine structure constants of ^85^Rb and ^87^Rb were detected as a function of the droplet size. In this theoretical follow-up article we try to explain and reproduce these observed shifts within a framework of relativistic ab initio calculations and a simulation of the helium droplet environment based on density functional theory.

In helium nanodroplet isolation spectroscopy^5^, ^6^ the alkali-metal atoms are particularly interesting as they stay on the surface of the superfluid He droplets, whereas most other atoms or molecules move to the center. For the alkali-metal atoms and their diffuse s electron the compensation of Pauli repulsion and van der Waals interaction forces leads to the formation of a “dimple” on the droplet surface in which the alkali-metal atom resides. Common methods employed to estimate the size and shape of these pockets, i.e. the resulting helium density distribution of such a weakly bound system, are density functional theory (DFT) or quantum Monte Carlo (QMC) approaches. A detailed study of dimple structures for alkali-metal-doped ^3^He and ^4^He droplets based on the latter is given in ref. 7. We will compare the results obtained with our method of choice, a time-independent DFT approach using the Orsay–Trento functional^8^ to map the He density onto the total energy, to the published QMC solvation energies. Previous studies mainly focused on the weak interaction between the dopants and the helium droplet and on those properties that can be derived from the variation of the total energy as a function of the geometry, for example, as a function of the distance between the dopant and the center of mass of the helium droplet. In this article, however, geometric effects on the total electronic wavefunction of the dopant are particularly interesting to us. Unfortunately, the problem of finding a computationally feasible bidirectional description of the coupling between the helium density distribution and the electronic wavefunction of the dopant has not yet been solved. Methods that in principle can account for dynamic effects such as the coupling between vibronic modes of the dopant and excitations of the helium droplet are diffusion or path integral techniques^9^, ^10^ or hybrid strategies, where the latter are combined with standard quantum chemistry approaches based on molecular orbital theory.^11^–^14^ Recently, the combination of time-dependent DFT with Bohmian dynamics has also been suggested.^15^ Despite this ongoing work there is still a huge technical gap between the two core aspects of simulating helium-droplet-induced ESR shifts: 1) A good description of the He density is needed, which includes all intrinsic quantum mechanical properties of the system but still scales reasonably with the system size. The first condition impedes the usage of molecular mechanics models, which are more likely to be applied to crystal-like clusters of larger rare gases such as Ar or Xe, but not to a superfluid quantum liquid such as a ^4^He droplet. The second condition, a reasonable scaling with the size of the system, prevents us from using highly sophisticated standard methods of MO-based quantum chemistry. 2) Accurate predictions of ESR properties can hardly be achieved without the application of such MO-based quantum chemistry techniques.

Hence, mediating between these frontiers, we come up with a simple but effective combination of a time-independent, well-established DFT approach for the description of the helium density with a powerful ab initio post-Hartree–Fock method for the simulation of the electronic wavefunction of the dopant. Our article is structured as follows: In Section 2 we give a brief introduction to the origin of the hyperfine splitting in alkali-metal atoms, discuss the set of simplifications applied to obtain estimates for the ESR shifts, and elaborate on both parts of the strategy mentioned above, namely the He DFT and the ab initio part, in two separate subsections. A third subsection is dedicated to the connection of both aspects, linking the obtained dimple profiles to changes in the total electron wavefunctions of the dopants. Results are then presented in Section 3 and compared to experimental data that are available for Rb-doped He droplets at least. We summarize our work in Section 4.

## 2. Theory

The interaction between the magnetic dipole moment *μ*_e_ of the electron and *μ*_n_ of the atomic nucleus gives rise to a hyperfine structure (HFS) in the electronic energy levels. A special case concerns the ground state of atoms with a single valence electron in an s-type orbital, which is only affected by the isotropic part of this interaction. Assuming that the quantum number *I* for the nuclear spin and *J* for the total angular momentum of the electronic wavefunction are good quantum numbers, the relative change in energy due to hyperfine interaction is given by Equation (1):


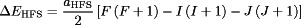
(1)

with *F*=*I*+*J* denoting the total angular momentum of the atom, and *a*_HFS_ the hyperfine splitting constant. As noted by Fermi, the latter may be written as Equation (2):


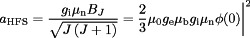
(2)

with *g*_e_, *g*_i_, *μ*_0_, *μ*_b_, *μ*_n_ and |*ϕ*(0)|^2^ as the electron spin *g* factor, the nuclear spin *g* factor, the vacuum permeability, the Bohr magneton, the nuclear magneton and the electron spin density at the nucleus, respectively. This contribution to the hyperfine structure is historically known as the Fermi contact term. In ref. 16 the usage of “Fermi penetration term” is suggested to emphasize that it arises from the field of the magnetic dipole moment of the electron inside the finite volume of the nucleus.

Our approach to the simulation of HFS shifts for alkali-metal atoms attached to helium droplets consists of four steps: 1) The selection of appropriate all-electron basis sets for the alkali-metal atoms from Li to Rb. 2) The calculation of the ^2^Σ_1/2_ ground states of the He*M* (*M*=Li,Na,K,Rb) diatomic systems, providing the binding energy and the spin density at the alkali-metal atom, |*ϕ*(0)|^2^, as a function of the He–M distance. Equation (2) maps each obtained density onto a corresponding hyperfine splitting constant *a*_HFS_. 3) A DFT simulation of alkali-metal atoms attached to different droplet sizes with *N*, the number of helium atoms, ranging from 50 to 10 000, using the diatomic potential energy curves of step 2 as input. From the obtained density profiles we evaluate average distances between the dopant atom and its direct helium neighborhood. 4) The information gained from step 3 on the functional relationship between averaged He–Rb distance and droplet size is combined with the relationship from step 2 between He–Rb distance and spin density, allowing a prediction of ESR shifts on helium droplets for all four alkali-metal atoms.

The size of the He_*N*_Rb clusters and the fact that these systems are held together by weak dispersion forces keeps these objects out of the range of current wavefunction-based quantum chemistry approaches. The spin density at the nucleus, on the contrary, is a quantity which can be rather easily derived from these methods. Hence, linking quantum chemistry to a less costly description of the cluster is highly desirable, but inevitably entails a list of simplifications, leading to systematic errors of different magnitude. The first two steps refer to the application of post-Hartree–Fock methods. Intrinsic errors of these methods derive from the usage of a finite basis and the incomplete recovery of correlation energy due to limitations of the chosen methods. For our choice of basis set, the former is negligibly small compared to the latter. However, all inaccuracies at the ab initio level are small compared to the simplifications made in step 3, where we assume the electronic wavefunction of the alkali atom to be fully static during the DFT-based simulation of the relaxation on the helium droplet. This is an intrinsic limitation of the DFT code, where the dopant–droplet interaction is accounted for by an integration over fixed pair potentials, which prevents us from a direct analysis of droplet-induced changes of the electronic wavefunction. The response effect of such an electronic perturbation on the current He density, on the other hand, is expected to be negligibly small. From the DFT results for the He density distribution we estimate the mean distance between the alkali-metal atom and helium environment. Finally, in step four, we look at the He_*N*_–M system as an effective diatomic system,^17^–^19^ and relate the magnitude of the HFS splitting to the droplet size.

### 2.1. Ab Initio Part: Spin Densities of *M*–He at the Alkali Nuclei

A series of M–He (*M*=Li,Na,K,Rb) diatomic systems were calculated using the normalized elimination of the small component (NESC) method^20^–^23^ in connection with the coupled-cluster singles and doubles (CCSD) formalism. The hyperfine constant is obtained using the NESC analytic derivatives formalism^24^–^26^ combined with the relaxed density matrices from CCSD. In the NESC calculations, a Gaussian finite-size nucleus model was used on all atoms with the nuclear charge radii taken from the compilation of Dyall and Visscher.^27^ When calculating the hyperfine structure (HFS) constants,^26^ a finite distribution of the nuclear magnetic moment modeled by a Gaussian function was used. Because the experimental values of the magnetic nuclear radii are not available for most of the elements, the nuclear charge radii were employed instead. The open-shell species were calculated using the spin-unrestricted formalism. All electrons were correlated in the CCSD calculations.

The following basis sets were used in the calculations. For helium, the aug-cc-pVQZ-DK basis set^28^, ^29^ was used in all the calculations. For lithium, the cc-pVQZ-DK basis set^29^, ^30^ was combined with the core–valence correlating functions from the cc-pCVQZ basis set.^31^ One tightmost s-type primitive function was decontracted and the basis set was augmented by three tight s-type primitive functions in a geometric sequence. The resulting (18s9p5d3f1g)/[12s7p5d3f1g] basis set provides for the calculated isotropic HFS constant which remains unchanged (within ca. 0.1 MHz) by adding further tight basis functions. The (25s15p6d4f2g)/[12s8p6d4f2g] basis set for sodium was constructed in a similar way using the cc-pVQZ-DK and cc-pCVQZ basis sets augmented by three tight s-type primitive functions in a geometric sequence. For potassium and rubidium, the relativistically optimized quadruple-zeta-quality basis sets of Dyall^32^ were used. The original basis sets were augmented by a single tight s-type basis function. The resulting (32s22p6d4f2g)/[15s13p6d4f2g] (K) and (36s26p19d7f4g)/[16s14p10d7f4g] (Rb) basis sets provided for the isotropic HFS constant values converged with respect to further basis set extension. For comparison of the calculated isotropic HFS constants with the experimental data and reference calculations from the literature see Table 1.

**Table 1:** NESC/CCSD isotropic HFS constants [MHz] for alkali metal atoms in the ground electronic state in comparison with the experimental values and the reference literature data

Atom	Exp.^33^	NESC/CCSD	DC-CISDpT[a]
^7^Li	401.7520433(5)	402.0	–
^23^Na	885.8130644(5)	880.2	888.3
^39^K	230.8598601(3)	232.1	228.6
^85^Rb	1011.910813(2)	1019.1	1011.1

[a] Relativistic four-component Dirac–Coulomb configuration interaction singles and doubles with perturbative triples from ref. 34.

**Figure 1:**
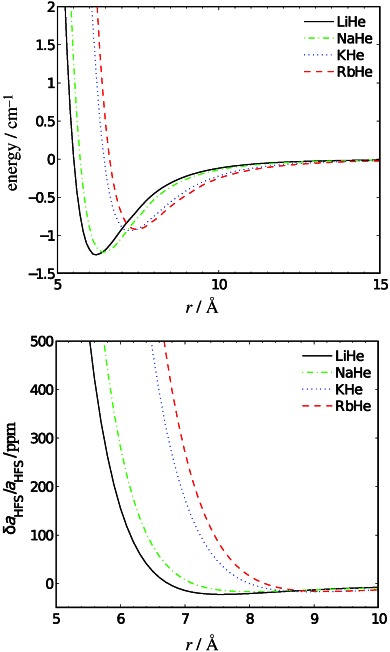
Top: Potential curves of the X^2^Σ_1/2_ states of the M–He (*M*=Li, Na, K, Rb) diatomic systems. The binding energy decreases with growing size of the alkali-metal atoms, the position of the minimum shifts to larger distances. Bottom: Relative shift of the HFS constant *a*_HFS_ as a function of the interatomic distance. Beside its drastic increase at small distances, a slight quenching can be observed in the minimum-energy region due to the polarization effects on the valence s-shell of the alkali-metal atoms.

Our results for the dependence of the HFS constant on the interatomic distance at the NESC/CCSD level of theory are summarized in Figure 1, together with the potential energy curves of the corresponding X^2^Σ_1/2_ electronic ground states of the diatomic systems. For the latter we employ a single-reference^35^ partially spin-restricted open-shell variant of the coupled-cluster method with single and double excitations plus perturbative triples [RHF-RCCSD(T)],^36^, ^37^ which is implemented in the Molpro program package.^38^ This allows us to obtain slightly higher amounts of the correlation energy. For K and Rb, scalar relativistic corrections were taken into account on the basis of the second-order Douglas–Kroll Hamiltonian.^39^, ^40^ For Li, Na and K all electrons were correlated, for Rb the neon core was kept frozen. All potential energy curves were corrected for the basis set superposition error using the counterpoise method of Boys and Bernardi.^41^, ^42^

We note that our potential curves differ slightly from the commonly used ones of Pascale^43^, ^44^ or Patil.^45^ The former suggest significantly deeper minima than we obtained (between 2.5 cm^−1^ for LiHe and 1.3 cm^−1^ for CsHe), while the latter, which are based on second-order exchange perturbation theory with damped van der Waals interaction terms, are in good agreement with our results. In general, they show only slightly deeper potential minima and longer binding distances (except for RbHe). A direct comparison is given Table 2.

**Table 2:** Binding energies [cm^−1^] and minimum-energy distances [Å] for the X^2^Σ_1/2_ states of the *M*–He diatomic systems

	LiHe	NaHe	KHe	RbHe
Δ*E*	−1.256	−1.220	−0.935	−0.918
Δ*E* ^[45]^	−1.33	−1.20	−0.98	−0.98
*r*_min_	6.160	6.404	7.133	7.376
*r*_min_^45^	6.197	6.408	7.181	7.334

The functional dependence of the HFS constant on the binding distance (Figure 1, lower graph) is similar for all alkali-metal atoms. A significant increase of *a*_HFS_ is observed for all species at short distances. This behavior is explained by the compression of the s-orbitals due to Pauli repulsion from the doubly occupied helium shells.^4^, ^46^, ^47^ Tentative scans of even shorter distances (not shown in the graph) prove that *a*_HFS_ approaches a maximum value at a certain distance before it drops down rather steeply, which can be explained by a strong intermixing of the s orbitals with orbitals of higher angular momentum. The same functional dependence of *a*_HFS_ on the internuclear distance, especially its collapse at very short binding lengths, was found earlier in refs. 48 and 49, where the trapping of hydrogen and alkali-metal atoms in rare-gas matrices (Ne, Ar, Kr and Xe) was simulated on the basis of diatomic potential energy surfaces. We note that the short-distance area is not relevant for our purpose but could become interesting for studies on systems of highly pressurized bulk helium. More relevant to our case is that all four diatomic systems show a reduced HFS constant around the minimum energy distance, which is due to polarization effects on the s-orbital of the alkali-metal atoms. This effect must be related to the amount of electron correlation energy and hence to the binding strength of the van der Waals-bound systems. As expected, the HFS constant decreases with the increasing binding energies from RbHe to LiHe.

### 2.2. DFT Part: Alkali Atoms on Helium Droplets

To account for a one-sided interaction between dopant and He droplet we apply a He density functional approach to obtain the free energy and density distribution for doped droplets of varying size. The Orsay–Trento density functional^8^ is used to map the He density onto the energy. We apply the FORTRAN code of F. Dalfovo with modifications of K. K. Lehmann and R. Schmied.^52^ It minimizes the free energy of a doped He droplet with respect to the dopant position, calculating a cylindrically symmetric equilibrium density distribution on a grid of cylinder coordinates *z*×*r*. A grid of 601×300 points with a spacing of 0.2376725 Å has been chosen for our purpose. The free energy *F*[*ρ*], a functional of the He density *ρ*, may be written as Equation (3):^53^


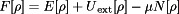
(3)

where *E*[*ρ*] denotes the Orsay–Trento functional and *U*_ext_ the external potential, which introduces the dopant interaction. The latter is based on the corresponding M–He ab initio pair potential from the previous subsection. This is done in order to keep our mixed approach as consistent as possible. A third term occurs in Equation (3) due to the constraint of a conserved number of He atoms. The particle number [Eq. (4)]:


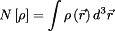
(4)

is multiplied by its corresponding Lagrange parameter, the chemical potential *μ*.

First, we look at the solvation energies for all alkali-metal dopants as a function of the droplet size. The solvation energy is defined as the energy difference between the doped and the undoped cluster, given by Equation (5):


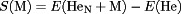
(5)

**Figure 2:**
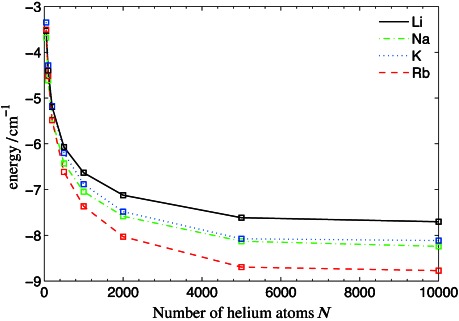
Solvation energies of Li, Na, K and Rb as function of the He droplet size.

The term “solvation energy” might be slightly misleading since the alkali-metal atoms are not solvated in the droplet but attached to its surface. However, the above definition is still valid and its nomenclature is also commonly used for the droplet-to-dopant binding energy. Our results are summarized in Figure 2. The absolute values of the solvation energies increase with the size of the droplet, and are slightly higher for the larger alkali metal atoms. Our values are in good agreement with the QMC results of ref. 7.

### 2.3. A Model for ESR Shifts Induced by He Droplets

**Figure 3:**
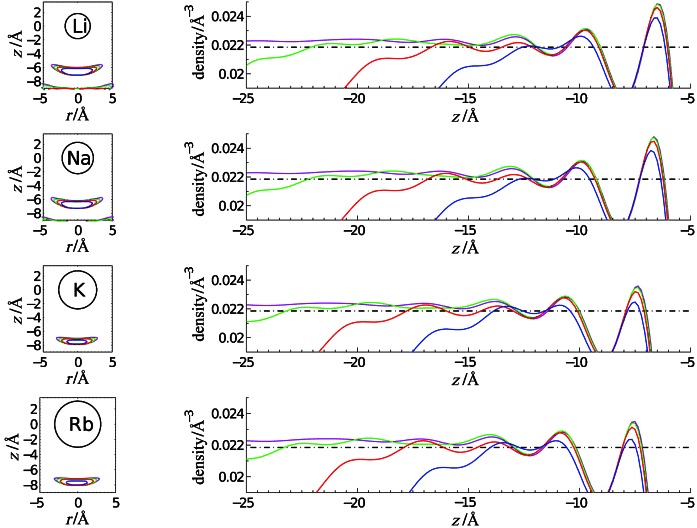
Left column: Contour plots of the helium density obtained for He droplets of *N*=50, 100, 200, and 500 and the four different alkali-metal dopants. Note that each contour line represents the same density value (*ρ*=0.02185 Å^−3^) but corresponds to a different He droplet size. The innermost contour line is obtained for the smallest, the outermost for the largest He droplet. The alkali-metal atoms are plotted according to the van der Waals radii taken from refs. 50 and 51. Right column: Radial He density along the “internuclear” axis going through the center of mass of the He density and the alkali-metal atom, plotted for different He droplet sizes (blue, red, green and magenta for *N*=50, 100, 200 and 500). The alkali-metal atom is located at *z*=0. Note the fluctuation of the He density, showing a global maximum between −5 and −8 Å. Larger droplets are omitted, because their profiles are practically indistinguishable from the He_500_ droplet in the relevant area around the first density maximum at this scale. The bulk density is shown as a dash-dotted line for comparison. The corresponding contour plots (left column again) can be interpreted as cuts through the density landscapes along the dash-dotted line.

For the analysis of HFS shifts the He density distributions in close proximity to the dopants are more important than solvation energies. It is a well-known phenomenon that alkali-metal doped He droplets exhibit periodic fluctuations of the He density close to the dimple where the dopant resides. Graphs of the radial density along the “internuclear” axis connecting the center of mass with a heliophobic dopant can be found in Figure 3 (see right column), and are similar to those given in ref. 54. Here we analyze the change of these profiles with increasing droplet size, with special interest in the first and highest He density peak, which occurs 5 to 8 Å off the dopant position. Two essential features can be observed: first, the peak value increases with droplet size, and second, its distance to the dopant decreases with the droplet size. According to our results obtained for the M–He systems both of these tendencies suggest an increase in the HFS constant for larger droplets. This simplified diatomic-like picture is supported by the results we obtain for the two-dimensional plots of the He density distribution. In the left column of Figure 3 we present He density contour lines of the same density value (the bulk helium value *ρ*=0.02185 Å^−3^), one for each droplet size, proving that the first maximum of the density is surprisingly well localized. Each banana-shaped contour line in the two-dimensional cut corresponds to a small, saucer-shaped area of high He density in the real droplet. Since it lies closest to the droplet, the assumption of it being the main reason for HFS shifts is self-suggesting. Larger droplets show larger contour rings as the He density peak grows, which move also closer to the dopant with increasing droplet size. This suggests that information on the size and position of the compressed He density can be related to the droplet-induced change of the HFS constant.

An alternative way of approximating the relative shift of *a*_HFS_ of a dopant in a rare-gas matrix on the basis of single-atom contributions (also derived from ab initio calculations on diatomic systems) has been suggested in ref. 48, where the dopant was embedded in a crystal lattice of rare-gas atoms as a substitutional or interstitial impurity. The relative shift of *a*_HFS_ was then obtained by a summation over all relative shifts induced by each rare-gas atom. Here we extend this method to continuous densities by integrating the product of the diatomic functions *a*_HFS_(*r*) of Figure 1 (bottom) and the He density of each droplet over the total volume [Eq. (6)]:


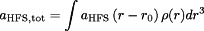
(6)

with *r*_0_ denoting the position of alkali-metal atom. This method reproduces the ordering and also leads to a functional dependence similar to that shown in Figure 5. However, it overestimates the the shift of *a*_HFS_ by a factor of about 2.5. For the smallest droplet size we obtain shifts of 211, 303, 370, 428 ppm, for the largest we obtain 529, 762, 1076 and 1270 ppm for Li, Na, K and Rb, respectively. Furthermore, convergence towards droplet size is slower than in the preferred model described above. Although this summation technique could be successfully applied to the theoretical analysis of alkali-metal trapping sites in rare-gas matrices (Ne, Ar, Kr, Xe),^49^ its application to our case seems to be less fruitful. We give two reasons for this failure: first, there is no clear reason to assume that the shift of *a*_HFS_ induced by several rare-gas atoms adds linearly to the total effect. The argument given in ref. 48, saying that the error in energy caused by neglecting three-body interactions is small, hence the same is to be expected for the shift of the HFS constant, is not very strong. The functional dependence is not known, and its direct transferability to the special case of liquid He nanodroplets is questionable. Second, a pair model description cannot fully account for non-isotropic distortions of the electronic wave function of the dopant. We note that the latter issue is of minor relevance in the bulk (or in a periodic grid of rare-gas atoms), it even vanishes for single atoms residing in the center of a He droplet, but is highly relevant in the case of surface deformations such as the dimple environment.

A last but cumbersome alternative would be the direct evaluation of geometry-dependent three-body contributions to the shift of *a*_HFS_. However, such an approach lies beyond the scope of our article.

## 3. Results and Discussion

**Figure 4:**
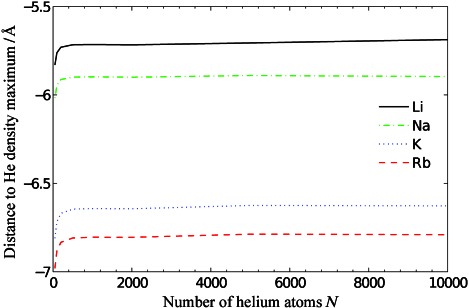
Distances between the point of maximum He density along the internuclear axis and the dopant as a function of the He droplet size. Note that the alkali-metal atoms reside above the droplet at *z*=0 (see Figure 3), hence the negative values on the ordinate.

**Figure 5:**
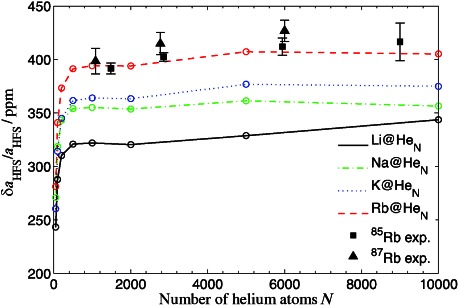
Relative shifts of the HFS constant *a*_HFS_ as a function of the He droplet size. Experimental results available for ^85^Rb (ref. 4) are shown as black triangles.

From the previous section we conclude that the distance between a certain He density contour line and the dopant position may be used to predict HFS shifts in He droplets. In a first attempt, we choose the position of the He-density maximum on the *z*-axis as key value (Figure 4), looking up the corresponding change of the HFS constant in the lower graph of Figure 1. However, the thus-obtained shifts are too small when compared to the available experimental measurements for Rb. Furthermore, the position of the first He-density peak seems to be converged already at cluster sizes between 500 and 1000. In a second approach we choose a contour value (*ρ*=0.0174 Å^−3^) that reproduces the experimental ^85^Rb shift at a droplet size of *N*≍2000. With this choice of parameter we obtain results in good agreement with the available experimental data for ^85^Rb and ^87^Rb,^1^, ^2^ confirming the suggested 1/*N* trend of the functional dependence. They are plotted together with our predictions for Li, Na and K in Figure 5. The relative change of *a*_HFS_ is the same for both Rb isotopes. The absolute value of *a*_HFS_ for atomic ^87^Rb can be obtained from Table 1 by rescaling the ^85^Rb value with the nuclear *g*-factor ratio of 3.38898.^55^ From Figure 5 it can be seen that our model predicts, at least for Na, K and Rb, the convergence of the HFS shifts to the asymptotic value at a droplet size of about *N*=10 000. Ref. 1 also reports a measured shift of 325±40 ppm for the HFS constant of ^39^ K at *N*=8000, which is in reasonable agreement with our prediction.

The curves for Li, Na and K show a similar functional dependence of *a*_HFS_ on the droplet size, although the HFS shifts are less pronounced than those for Rb. All of them are positive, although polarization effects, which play a bigger role for the lighter alkali-metal atoms (see Figure 1), lead to lower values for *a*_HFS_ in the M–He diatomic systems at the minimum-energy distance. However, the binding distances for all alkali-metal atoms attached to He droplets are significantly shorter than the internuclear distances of the diatomic potential minima. All diatomic systems show an almost exponential increase of *a*_HFS_ in the relevant region between 6 and 7 Å. The steepness of the curves grows with increasing size of the atom, explaining the stronger shift of *a*_HFS_ for Rb compared to that for Li on He droplets.

We note that our simple approach fully neglects the fact that the alkali-metal atoms are surrounded by several helium atoms corresponding to the diffuse helium density distribution in the direct vicinity of the dopant. Furthermore, it does not account for any changes in the dimple geometry beside those covered by the single parameter we have decided to use as indicator. From the left column of Figure 3 it can be seen that the area of compressed helium also starts to encompass the dopant with increasing droplet size. The omission of this additional effect can possibly explain the early convergence of our model description, which seems to be challenged by the experimental results for clusters beyond our size limit, which are still showing a marginal increase for He droplets with *N*=15 000.^4^ Nevertheless we believe that our model, which is based on a single empirical parameter applied to all alkali-metal dopants, can be of value for future ESR experiments on doped He droplets.

## 4. Summary

We predict He-droplet-induced changes of the isotropic HFS constant *a*_HFS_ of the alkali-metal atoms M=Li, Na, K and Rb on the basis of a model description. He density distributions of droplet sizes ranging from 50 to 10 000 He atoms, obtained from He density functional theory, are mapped onto shifts observed for the HFS constant in the M–He diatomic systems as a function of the interatomic distance. This dependence was calculated at the CCSD/NESC level of theory with basis sets of quadruple-zeta quality, augmented by tight s-functions to obtain the necessary accuracy in the core region. The link between the ab initio approach and density functional theory is based on the observation that the maximum of the He density is well localized in close proximity to the dopant, sitting directly under the surface distortion caused by the heliophobic dopant residing on the droplet surface, commonly known as a dimple. We showed contour plots of the He density and radial density profiles to support our simple model approach. Good agreement with experimentally measured changes of *a*_HFS_ in the case of ^85^Rb could be achieved by measuring the smallest distance between the alkali-metal atom and an arbitrarily chosen He density contour line at *ρ*=0.0174 Å^−3^. The approach is then used to make predictions for He-droplet-induced changes of the HFS constant for Li, Na and K.
